# Descriptive Epidemiological Analysis for the First Outbreak of Lumpy Skin Disease in Japan in 2024

**DOI:** 10.1155/tbed/8488125

**Published:** 2025-09-29

**Authors:** Yoko Hayama, Ryosuke Omori, Ryota Matsuyama, Sonoko Kondo, Emi Yamaguchi, Yuzu Kamata, Takehisa Yamamoto

**Affiliations:** ^1^Division of Transboundary Animal Disease Research, National Institute of Animal Health, National Agriculture and Food Research Organization, Tsukuba, Ibaraki, Japan; ^2^International Institute for Zoonosis Control, Hokkaido University, Sapporo, Hokkaido, Japan

## Abstract

Lumpy skin disease (LSD) is a transboundary emerging disease of cattle and water buffaloes that threatens the livestock industry globally. Japan experienced its first outbreak in November 2024. This study aimed to describe the spatial and temporal characteristics of this outbreak and estimate the transmissibility using a mathematical model for within-farm transmission. The first and second cases were confirmed on dairy farms in Itoshima City, Fukuoka Prefecture, southern Japan, on November 6, 2024. Twenty-two farms were confirmed during this outbreak, with 17 cases in Itoshima City and the other two municipalities in Fukuoka Prefecture. The third case occurred in Kumamoto Prefecture on November 8, 2024, and was linked to the long-distance movement of potentially infected cattle via the livestock market from the first case on October 18, 2024. Two additional cases were detected near the third case. Control measures included isolation and voluntary culling of infected cattle; voluntary movement restrictions on infected, suspected, and apparently healthy cattle on the same premises; and voluntary suspension of the raw milk and semen shipments from infected and suspected animals. These measures were voluntary; however, no violations were reported. Vector control was achieved with insecticides and insect-proof netting. Voluntary vaccination was conducted within a 20 km radius of affected farms in Fukuoka Prefecture. Mathematical modeling of within-farm transmission dynamics revealed a transmission rate of 0.0031 (95% CI: 0.002–0.0044) per day. The basic reproduction number was 3.51 (95% CI: 2.26–4.98) based on a herd size of 49 and an infectious period of 23.1 days. Although the outbreak was geographically limited, this study highlights key epidemiological features of LSD, including its high transmission rate and long-distance transmission via cattle movement. Japan has a persisting LSD virus (LSDV)incursion risk due to recent outbreaks in Asia. Strengthening preparedness, including awareness among farmers and veterinarians, emergency vaccination plans, vector control, traceability, and quarantine protocols for cattle movement, is essential to mitigate future outbreaks.

## 1. Introduction

Lumpy skin disease (LSD) is a transboundary emerging disease affecting cattle and water buffaloes. The causative agent, LSD virus (LSDV), is a double-stranded DNA (dsDNA) poxvirus from the genus *Capripoxvirus*. The primary clinical signs include nodules and swelling on the skin, fever, depression, enlarged lymph nodes, nasal discharge, tear shedding, reduced milk production, and lameness [1]. Transmission mainly occurs through biting insects such as flies, mosquitoes, midges, and ticks [1]. Non-vector-borne and indirect transmission, including sharing water troughs, has also been reported [2–4]. The mortality rate is usually less than 10% [5]. However, the disease results in reduced milk production and temporary or permanent infertility. LSD is designated as a listed disease by the World Organization for Animal Health (WOAH) due to its significant economic impact through livestock production losses and international trade restrictions [6].

The global spread of LSD poses an international threat. LSD was first reported in Zambia in 1929 and subsequently became endemic in most countries in Africa. It has been spreading to Middle Eastern countries, including Turkey, since 2012 [7]. The first outbreak of LSD in Europe was reported in Greece in 2015, and the disease has spread to Eastern Europe [7]. Between 2010 and 2019, several vaccine-like recombinant strains of LSDV were discovered in Kazakhstan and neighboring regions of Russia and China [8]. The first novel recombinant LSDV strain was identified in Russia in 2017 [9] and subsequently in Kazakhstan in 2018 [10]. Since 2019, LSD has spread rapidly across Asia, with outbreaks reported in Bangladesh, India, and China [11, 12]. Between 2020 and 2022, additional cases were documented in Chinese Taipei, Myanmar, Bhutan, Hong Kong, Nepal, Sri Lanka, Vietnam, Laos, Mongolia, Malaysia, Cambodia, Thailand, Singapore, and Indonesia [11, 13–19]. In the Republic of Korea, an outbreak occurred in October 2023, and prompt nationwide vaccination successfully contained the disease by December 2023 [20]; however, LSD reemerged in August 2024 [20]. Japan remains vigilant against potential LSDV incursions owing to its recent emergence in neighboring countries, including the Republic of Korea and China.

Understanding the transmission dynamics of an infectious disease is critical for implementing effective control measures. Mathematical modeling provides a useful approach for characterizing the spread of infectious disease. Specifically, the basic reproduction number (*R*_0_), defined as the average number of secondary cases generated by a single infected individual in a fully susceptible population, is a key parameter for quantifying disease transmissibility. However, empirical data on within-farm transmission of LSD and estimates of *R*_0_ remain limited.

The first outbreak of LSD occurred in Japan in November 2024. This study aimed to conduct a descriptive epidemiological analysis to investigate the spatial and temporal characteristics of this outbreak. It also aimed to estimate the transmissibility of LSD in cattle using a mathematical model informed by within-farm LSD transmission data.

## 2. Materials and Methods

### 2.1. Data and Descriptive Analysis

Data on the farms with outbreaks were provided by the Ministry of Agriculture, Forestry, and Fisheries (MAFF) and the affected prefectural governments. The data included information on herd size, type of farm (dairy, beef, or mixed), date of detection of clinical signs, date of notification, date of laboratory confirmation, number of animals with clinical signs, and reports from epidemiological investigations. We examined the course of the outbreak and control measures implemented for each affected farm. The spatial and temporal trends in the affected area were subsequently overviewed.

### 2.2. Estimation of Within-Farm Transmissibility of LSD

The data from the third case farm (Case 3) were used to estimate the transmissibility of LSD. This farm was selected because the date of disease introduction was clearly identified based on the known movement of a potentially infected animal from the first case farm (described later). The dates of clinical onset and culling were recorded for all animals, providing an appropriate dataset for epidemiological modeling. Data from other affected farms were not used owing to incomplete information on clinical onset or culling dates, or because the number of affected animals was too small for reliable analysis. On this farm, among the 49 adult cattle, 25 (51%) developed clinical signs, including characteristic skin nodules, and were subsequently culled. Data for all 49 animals were included in the dataset. Vaccination was not conducted on this farm.

We estimated the basic reproduction number (*R*_0_) to quantify transmissibility. It represents the expected number of secondary cases from an index case at the beginning of an outbreak. We assumed the number of vectors for LSD transmission was constant over time because the duration of the outbreak at the farm was short enough to neglect a seasonal effect. The constant transmissibility of an infected host allows us to express *R*_0_ as

(1) $$\begin{array}{c} R_{0}=\beta S_{0}D, \end{array}$$

where *β* denotes the transmission coefficient, *S*_0_ denotes the number of susceptible hosts when the index case was introduced, and *D* denotes the length of the infectious period. *β*, *S*_0_, and *D* are required to estimate *R*_0_. Based on a previous experimental infection study that estimated the infectious period via the detection of virus or viral DNA in skin lesions [21], we assumed *D* = 23.1 days. Regarding *S*_0_, we set 49 as the number of cows on the farm that were likely to contract an LSD infection based on the actual records of the outbreak. *β* was estimated from the data on the timing for clinical onset and culling at individual level. We developed a mathematical model of infection and disease progression based on several assumptions: (i) infection risk for a susceptible cow is proportional to the number of cows with clinical onset, (ii) the timing of clinical onset coincides with the beginning of the infectious period, and (iii) the waiting time from infection to clinical onset follows a gamma distribution. Using these assumptions, force of infection at time *t*, *λ*(*t*), can be written as

(2) $$\begin{array}{c} \lambda \left(t\right)=\beta I\left(t\right), \end{array}$$

where *I*(*t*) denotes the number of cows showing clinical onset at time *t*. Using *λ*(*t*), the likelihood of clinical onset for the *i*-th cow at time *T*_*i*_, *L*_infected,*i*_, can be written as

(3) $$\begin{array}{c} L_{\text{infected,}i}=\int_{\tau =0}^{T_{i}}\exp\left[-\int_{u=0}^{T_{i}-\tau }\lambda \left(u\right)du\right]\left(1-\exp\left[-\lambda \left(T_{i}-\tau \right)\right]\right)\text{pdf}\left(\text{ga}\left(k,\theta \right),\tau \right)d\tau , \end{array}$$

where *T*_*i*_ represents the observed time of clinical onset for the *i*-th cow, and pdf(ga(*k*, *θ*), *τ*) denotes the probability density function of gamma distribution with shape parameter *k* and scale parameter *θ* for observing the waiting time from infection to clinical onset *τ*. Based on a previous study [21], we assumed that the durations from infection to clinical onset follow a gamma distribution with a mean of 7.3 days and a variance of 2.1 days. Meanwhile, the likelihood that the *i*-th cow was not infected during the study period, *L*_susceptible,*i*_, is given as

(4) $$\begin{array}{c} L_{\text{susceptible,}i}=\exp\left[-\int_{u=0}^{\hat{T}}\lambda \left(u\right)du\right], \end{array}$$

where $\hat{T}$ denotes the time at the end of study period (i.e., the day when all animals showing clinical onsets were culled). The likelihood based on the entire record of clinical onset times can be

(5) $$\begin{array}{c} L=\prod_{i}L_{\text{infected,}i}\prod_{j}L_{\text{susceptible,}j}. \end{array}$$

*β* was estimated to maximize *L*. The data was recorded daily, and *L*_i_ was also discretized in daily units during computation. *R*_0_ can be estimated by substituting the estimated *β* into Equation (1). The profile likelihood-based confidence intervals were also calculated for *β*. All computations were conducted using Mathematica ver. 14.0.0.0.

## 3. Results

### 3.1. Background of the Affected Area

**Figure 1:**
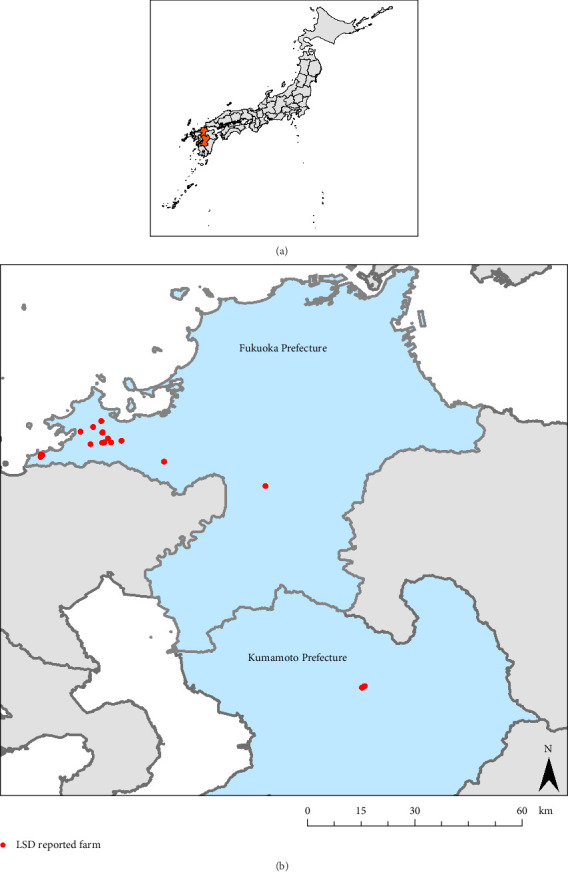
Affected regions during the lumpy skin disease outbreak (LSD) in Japan in 2024. (A) A map of Japan. The orange colored area represents the LSD-affected prefectures. (B) Enlarged map of the affected region.

LSD outbreaks were reported in Fukuoka and Kumamoto Prefectures, which are located in the northern part of Kyushu Island in Southern Japan (Figure 1). Kyushu Island is known for its thriving livestock industry. The southern part of the island is a major livestock farming area, especially for beef breeding and fattening cattle (Supporting Information Figure S1 and Table S1). Kumamoto is the most active region for dairy farming among the prefectures in Kyushu.

### 3.2. Course of Outbreak

The first (Case 1) and second cases (Case 2) of LSD were reported on November 5, 2024. Both were dairy farms with tie-stall housing that were raising 75 and 69 cattle, respectively. They were located in Itoshima City, northern Fukuoka Prefecture (Figure 1), and are 16 km apart.

On November 5, 2024, a private veterinarian visited these farms and found suspicious clinical signs of LSD, including skin nodules, enlarged lymph nodes, and fever in several cattle. The veterinarian subsequently reported to the local veterinary service center. Prefectural veterinary officers collected samples from blood, nasal swabs, and skin specimens from the affected cattle. These samples were sent to the National Institute of Animal Health (NIAH) for LSD diagnostic testing. The NIAH confirmed LSD through real-time and conventional polymerase chain reaction (PCR) on November 6, following the guidelines of the WOAH Terrestrial Manual [1]. The number of animals that developed clinical signs reached 48 (64%) on the first case farm and 33 (48%) for the second case farm. The phylogenetic analysis of the virus isolated from the first farm revealed 100% identity with the vaccine-like recombinant strains that have been circulating in neighboring countries since 2019 (submitted by Watanabe et al. [22]).

On November 8, 2024, the third case of LSD (Case 3) was reported in Kumamoto Prefecture, adjacent to Fukuoka Prefecture. This is a dairy farm with free-stall housing, raising 63 cattle. This farm is located 90 km from the first case farm (Figure 1). On November 6, a private veterinarian found skin nodules covering the body of one cattle and suspected enzootic bovine leukemia (EBL), a common disease among dairy cow in Japan, and requested a diagnosis testing for EBL to the local veterinary service center. On November 7, the local veterinary service center received a notification from the Livestock Division of Kumamoto Prefecture, which conveyed information from Fukuoka Prefecture regarding the transportation of cattle from the first case farm in Fukuoka Prefecture via a livestock market on October 18, 2024. The prefectural veterinary officers visited the farm that evening and instructed the isolation of the cattle introduced from Fukuoka Prefecture. The prefectural veterinary officers revisited the farm on November 8 for an inspection and found that the introduced cattle and two others had developed clinical signs suspected of LSD. LSD was confirmed through testing at the NIAH on November 9. Of the 49 adult cattle on the farm, 25 (51%) had nodules characteristic of LSD.

**Figure 2:**
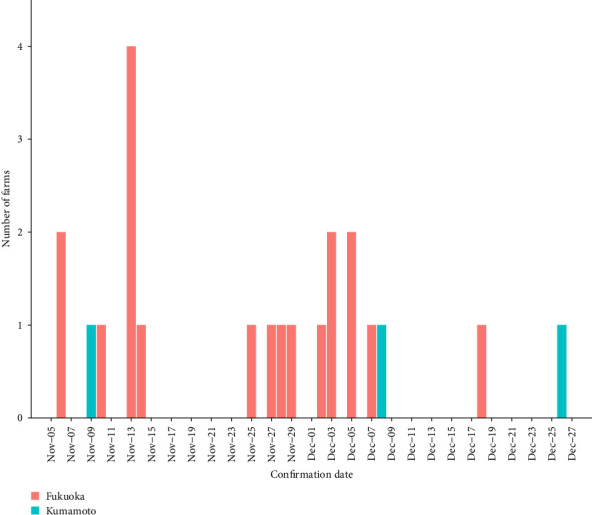
Temporal trend of the lumpy skin disease outbreak in Japan in 2024.

Following the confirmation of Cases 1 and 2 in Itoshima City, additional LSD cases were reported. Seventeen cases, including Case 1 and Case 2, within Itoshima City and the neighboring Fukuoka City by mid-December 2024 (Table 1, Figure 2). Among these, 12 cases were located within a 5 km radius and five cases within a 1 km radius. In total, fourteen were dairy farms, one was a mixed dairy and beef farm, and two were beef farms. In addition, two cases (Cases 13 and 18) were detected in dairy farms outside Itoshima City in Fukuoka Prefecture. Case 13 was detected on November 19, 2024, and Case 18 was detected on December 5, 2024. Case 13 was located in Nakagawa City, approximately 14 km from Case 1, and Case 18 was located in Asakura City, approximately 37 km from Case 1.

Two additional LSD cases (Cases 20 and 22) were reported in Kumamoto Prefecture after the confirmation of Case 3. Case 20, a mixed dairy-beef farm, was confirmed on December 8, 2024, and Case 22, a beef farm, was confirmed on December 26, 2024. Cases 20 and 22 were located approximately 780 and 250 m away from Case 3, respectively.

During this outbreak, 22 farms had LSD from November 2024 to the end of December 2024 (Table 1, Figure 2). Among these, 17 were dairy farms, three were beef farms, and two were mixed dairy-beef farms. The median herd size was 77 animals (range: 10–265). The median cumulative incidence for nodules typical of LSD was 5% per farm (range: 0.6%–64%). Farms where the disease was detected in November had a significantly higher median cumulative incidence of 15% (range: 2.3%–64%) compared with those detected in December (median of 1.5%; range: 0.6%–18.7%, *p*  < 0.001, Mann–Whitney *U* test). During the outbreak, the morbidity rate was 10.6% (230/2170). Eight cattle infected with LSD died during the outbreak; however, in seven of these cases, it was not confirmed whether LSD infection was the direct cause of death.

### 3.3. Control Measures

LSD is designated as a “Notifiable Infectious Disease” under the Act on the Prevention of Infectious Diseases in Livestock in Japan. This designation requires only monitoring, and no legally mandated control measures, such as culling infected animals, are enforced.

To address LSD outbreaks, MAFF established the “Guideline for Control and Prevention Measures Against Lumpy Skin Disease” in January 2024 [23]. This guideline recommends the following control measures for implementation on affected farms: (1) isolation and voluntary culling of infected cattle; (2) voluntary movement restrictions for infected, suspected, and apparently healthy cattle kept on the same premises; (3) voluntary suspension of raw milk and semen shipments from infected or suspected animals; (4) vector control using insecticides, repellents, and insect-proof netting; (5) voluntary vaccination in designated areas; (6) proper composting of manure and bedding; and (7) trace-back and trace-forward investigations of animals, people, and vehicles within 35 days and of semen within 42 days. These measures are not legally enforceable, but they are strongly recommended and implemented under the instruction of local veterinary officers based on the guideline.

The official veterinarians from the local veterinary service center visit the farm and take samples from blood, skin nodules, and nasal swabs (if available) when a suspected LSD case is reported. These samples were initially sent to NIAH for PCR testing during the early phase of the epidemic. However, the testing was conducted at the local veterinary service centers when the required systems were installed. A farm is designated as affected and subject to movement restrictions if the result is positive. Animals identified as clinically affected by the farmer or veterinarians on such farms are treated as infected and isolated, and the shipment of raw milk from them is voluntarily suspended. The restriction period for infected or suspected cattle and their milk is extended until (a) skin lesions disappear as confirmed by a veterinary officer or (b) results from a PCR test of a blood sample conducted 28 days after detection is negative. Movement restrictions for clinically healthy animals kept on the same premise are imposed until: (a) 28 days have passed since the last infected or suspected case was detected on the farm, or (b) 3 weeks have passed since LSD vaccination was implemented on the farm. Semen is withheld on the farm until a negative PCR result is confirmed 48 days after detection of the infected or suspected cattle. In addition, infected cattle on affected farms are subjected to voluntary culling based on the decision of the farmer and guidance from veterinary officers. Voluntary culling of infected cattle was carried out on 20 of the 22 affected farms during the outbreak, resulting in the removal of 90 animals.

The MAFF imported and stockpiled the live attenuated vaccine Lumpyvax (MSD Animal Health, Republic of South Africa) in July 2024 in response to the LSD outbreaks in the Republic of Korea. Vaccination is not compulsory, but prefectural governments recommend it to cattle farms within a 20 km radius of affected farms. They have planned or considered implementing vaccination campaigns in accordance with the LSD guidelines. The MAFF centrally manages the vaccine stock and distributes it to prefectural governments based on their vaccination plans. Vaccination is provided at no cost under the supervision of local veterinary authorities with the consent of farmers. Movement of cattle is restricted for 3 weeks following vaccination. As of the end of March 2025, 7543 animals on 241 farms in Fukuoka Prefecture had been vaccinated. However, no vaccination had been conducted in Kumamoto Prefecture, as voluntary culling of infected animals had been implemented on all three infected farms.

### 3.4. Estimation of Within-Farm Transmissibility of LSD

Within-farm transmission dynamics were analyzed using the outbreak data from Case 3. The estimated transmission rate between cattle was 0.0031 (95% CI: 0.002–0.0044) per day. Based on this estimate, *R*_0_ was 3.51 (95% CI: 2.26–4.98), assuming a herd size of 49 and an infectious period of 23.1 days.

## 4. Discussion

This study described the spatial and temporal characteristics of the first LSD outbreak in Japan in 2024 and the control measures implemented in the affected area. The transmission rate was estimated from a time series of clinical onsets and culling on a farm. While some experimental studies have examined vector-borne transmission parameters to cattle [21, 24], analyses of transmission parameters based on field data are limited. Therefore, our findings contribute to a better understanding of LSD transmission dynamics under field conditions.

Since 2019, LSD has rapidly expanded across Asia, with outbreaks reported in Bangladesh, India, and China [11, 21]. According to data from the World Animal Health Information System (WAHIS), several countries experienced large-scale outbreaks; Thailand reported 669 outbreaks between March 2021 and January 2024, Malaysia 322 outbreaks between May 2021 and January 2023, Vietnam 276 outbreaks between October 2020 and October 2023, and the Republic of Korea 107 outbreaks between October 2023 and February 2024. On the other hand, the 2024 LSD outbreak in Japan was limited in scale, with 22 cases. Concerning morbidity and mortality rates, in a previous study, it was reported that across six Asian countries, the average morbidity rate was 20.9% (ranging 9.52% in Myanmar to 37.1% in Thailand), while the average mortality rate was 2.68% (ranging from 0% in Laos and Myanmar to 7.7% in Vietnam) [25]. In comparison, the morbidity and mortality rates observed during the outbreak in Japan were relatively low. Variations in outbreak magnitude and disease impact across countries may be partly explained by contextual factors influencing disease spread, such as climate conditions affecting vector activity (temperature and humidity), cattle density, vector control implementation, and livestock movement systems. Cross-country comparisons should also account for differences in surveillance systems, case definitions, and notification requirements, which can influence how outbreaks are detected and reported. Given such heterogeneity in livestock production systems across the region, it is crucial to tailor surveillance and control strategies to each country's specific context, while continuing to monitor the dynamics of LSD spread in the region.

The 2024 LSD outbreak in Japan was limited in scale, with most cases concentrated in Itoshima City and a neighboring city in Fukuoka Prefecture. Of the 17 cases in this area, 12 occurred within a 5 km radius and five within a 1 km radius. A spatiotemporal analysis of the 2021 LSD outbreak in Thailand identified seven spatial clusters, and five of which had radii smaller than 5 km, with the largest cluster extending up to 10.6 km [26]. Another study used kernel-based transmission modeling in Thailand estimated the median between-transmission distance to be 0.3–0.8 km in Khon Kaen province and 0.2–0.6 km in Lamphun province, with 95% of transmission probability 5.2–40.4 km and 1.3–3.1 km, respectively [27]. Similarly, an analysis of the 2016 LSD outbreak in Albania indicated that most transmission occurs over short distances (<5 km), with 95% of transmission probability within 4.1–12.1 km [28]. These findings align with previous reports suggesting that, once introduced into a naïve region, LSDV spreads locally over short distances, with vector-borne transmission likely responsible for this pattern [2]. These findings highlight a consistent pattern of short-distance transmission across countries, emphasizing the need for rapid and localized containment measures, including movement restrictions, vaccination, vector control, and surveillance.

During the 2024 LSD outbreak in Japan, the farms with confirmed infections were concentrated in Itoshima City, Fukuoka Prefecture, with most of the infected farms being reported between early November and early December 2024. As of April 2025, no new infected farm has been reported since the last confirmed case at the end of December 2024. Epidemiological investigations identified a large number of stable flies (*Stomoxys calcitrans*) on affected farms in November 2024. Stable flies are potential vectors of LSDV, and recent experimental studies have highlighted their high transmission efficiency and ability to retain the infectious virus for at least 3 days [21, 24, 29–31]. They likely played a significant role in both within- and between-farm transmissions during the outbreak. The seasonal decline in stable fly activity during the winter may have contributed to the containment of the outbreak. Per previous studies, the optimal temperature for stable fly activity is approximately 21.8°C, with a threshold below approximately 10°C and above approximately 35°C [32, 33]. A field study conducted in Japan further indicated that stable fly activity declines at temperatures below approximately 12°C [34]. The average daily temperature reduced below 10°C in Itoshima City, and the maximum daily temperature remained below 12°C on most days from December 8, 2024 (Supporting Information Figure S2). These conditions likely suppressed stable fly activity, coinciding with the cessation of newly reported LSD cases and also a significant decline in the cumulative incidence on farms detected in December. Investigating the abundance and activities of stable flies on cattle farms, implementing stable fly control measures on farms, and managing manure practices that could serve as breeding sites for these vectors are important to better understand the role of vector activity in LSD transmission.

Although the first LSD case was confirmed in Fukuoka Prefecture on November 6, 2024, a potentially infected cattle was transported via a livestock market from the first case (Case 1) to the third case (Case 3) in Kumamoto Prefecture on October 18, 2024. The third case was later confirmed positive on November 9, 2024. Given the relatively long incubation period for LSD, which ranges from 2 to 4 weeks under field conditions [35] and 4–14 days under experimental conditions [36, 37], the virus was likely introduced to Japan in early to mid-October 2024. The source of the virus introduction into Japan cannot be identified. However, LSD reemerged in the Republic of Korea in August 2024, and outbreaks have been reported in other countries neighboring Japan during the same period [20]. The long-distance transport of insects by wind is considered a cause of outbreaks of agricultural pests or insect-borne plant and animal diseases [38, 39]. Strong wind patterns have been suggested as a possible mode for the introduction of LSDV-infected vectors, such as stable flies. For example, windborne transmission of infected vectors from Egypt to nearby Israel across distances of 80–447 km was hypothesized to have occurred during the LSD outbreak in 1989 [40, 41]. Stable flies can fly up to 29 km under laboratory conditions [42]. Field studies have shown that marked flies can be recaptured as far as 83 and 225 km from the point of release [41, 43]. Further investigation into the potential windborne incursion of the LSD-infected vector is warranted based on these precedents and the timing of outbreaks in nearby countries. Assessing the presence of vector species, wind patterns, and potential geographical points of origin will be critical to understanding the pathway of virus introduction.

Cattle movement poses a significant risk for long-distance transmission of LSD due to the long incubation period for LSD [35–37]. Per a recent study, cattle movement is more likely to contribute to the wide geographical spread of LSDV than mechanical transmission via vectors [44]. Long-distance transmission through cattle movement was confirmed during the LSD outbreak in Japan from Case 1 in Fukuoka Prefecture to Case 3 in Kumamoto Prefecture. Epidemiological investigations were conducted on affected farms to trace both backward and forward movements of cattle and related personnel and vehicles. The national cattle movement registration system facilitated the timely identification of cattle transported from an affected farm to other farms, enabling rapid implementation of control measures and likely limiting further spread of the disease. Cattle movement patterns in Japan have been analyzed at the regional level using the national cattle movement registration data. They showed that 82% of all between-farm movements for dairy cattle occurred within the same region (intra-regional), while 18% were between different regions (interregional) [45]. Similarly, 92% of all between-farm movements for beef cattle were intraregional, and 8% were interregional [46]. However, the cattle movement network at the prefectural, municipal, or farm-to-farm level has yet to be analyzed. Network-based analyses using movement data are necessary to identify high-risk areas for transmission and better assess the risk of LSDV spread through cattle movement.

Two cases (Cases 13 and 18) were confirmed in Fukuoka Prefecture in geographically separated areas located approximately 14 and 37 km away from Case 1, respectively. Epidemiological investigations revealed no cattle movements from the other infected farms. Stable flies can travel long distances when carried by strong winds [42, 43]. However, local dispersal of stable flies is generally limited to within 13 km in the absence of such conditions [47]. Most between-farm LSDV transmissions occurred over short distances (<5 km) likely due to localized vector dispersal during the LSD outbreaks in Israel in 2012–2013 and Albania in 2016 [5, 7, 28]. A similar pattern was observed in Thailand, where spatiotemporal analysis of the 2010 outbreak identified multiple clusters with radii less than 5 km, and kernel-based modeling estimated a median transmission distance of 0.2–0.8 km between farms [26, 27]. Specific meteorological conditions are required for long-distance windborne transmission of LSDV-infected vectors. Alternative routes of long-range transmission should also be considered, including inadvertent transport of vectors via human activities such as the movement of vehicles or farm equipment originating from affected areas (i.e., hitchhiker vectors) [48]. To prevent further spread, strict vector control should be implemented. According to the vector management guide for LSD control published by the Australian Department of Agriculture, Fisheries, and Forestry (DAFF) [49], a combination of environmental and chemical control measures is recommended. Environmental controls reduce suitable vector habits through practices such as removal of decaying manure or adjustments to grazing patterns, while chemical control using insecticides is commonly applied to livestock, vehicles, and equipment. Developing and implementing a context-specific vector control plan tailored to the environmental and management conditions of each cattle farm is crucial.

The estimated transmission rate between cattle was 0.0031 (95% CI: 0.002–0.0044) per day, and *R*_0_ was 3.51 (95% CI: 2.26–4.98), assuming a herd size of 49 and an infectious period of 23.1 days. Magori-Cohen et al. [50] estimated an indirect transmission rate (for vector-borne transmission) at 0.026 per day with an *R*_0_ of 15.7 per day during an LSD outbreak on a large dairy farm in Israel in 2006. In addition, a study from Ethiopia estimated transmission rates of 0.072 and 0.076 per day for the crop-livestock and intensive production systems, respectively, corresponding to *R*_0_ of 1.09 and 1.07 [51]. Several studies have attempted to estimate *R*_0_; however, it is worth noting that differences in modeling approaches and assumptions (e.g., herd size, production systems, or type of transmission considered) limit direct comparison across studies. Meanwhile, a modeling study based on experimental data demonstrated that stable flies (*S. calcitrans*) are likely the most efficient vectors of LSDV, with an estimated *R*_0_ of 15.5 [21] and 19.1 [24], higher than that of the other vectors such as biting midges and mosquitoes. These findings suggest that transmission of LSDV can occur at high rates, making outbreak control challenging when the LSDV has been introduced to the cattle population. Further, transmission rates may vary between farms depending on factors such as the abundance of stable flies at the time of detection, which is influenced by seasonal climate fluctuations, and spatial arrangements of cattle housing and manure storage areas. Further farm-level analyses are needed to identify the factors contributing to differences in transmission dynamics.

One limitation of the current study is the potential underreporting of affected cattle, especially for cases with mild clinical signs and nonvisible skin nodules. The MAFF and prefectural governments encouraged veterinarians and farmers to report suspected cases immediately after the first detection of LSD. However, some cases may have been missed, especially given that this was the first LSD outbreak in Japan and awareness and experience with the disease were limited. Epidemiological investigations, including backward and forward tracing of cattle movements, identified no additional suspected animals, except for Case 3. This suggests that the LSD outbreak remained geographically confined to limited areas within Fukuoka and Kumamoto Prefectures, without further spread to other regions. Not all infected animals develop clinical signs, and the incubation period can be relatively long. These factors may limit the feasibility of early detection based on clinical observation. Furthermore, the limited availability of entomological data, including vector abundance, activity patterns, and species composition, constrains the interpretation of transmission dynamics in this study. While vector-borne transmission is considered the most plausible mode of spread, the collection and analysis of vector surveillance data are necessary for a more detailed assessment of the role of specific vectors or environmental factors in shaping the outbreak.

Vaccination using live attenuated vaccines is considered the most effective method for controlling LSD. Early detection based on clinical signs is often difficult, and containment through culling alone may not be sufficient [52]. Mass vaccination campaigns against LSD in Southeastern Europe have underscored the strong field efficacy of live attenuated vaccines in eliminating disease spread. Coverage of up to 100% was achieved, leading to well-established herd immunity [5, 53]. In Asia, countries including Vietnam, Thailand, Malaysia, Cambodia, Indonesia, India, Bangladesh, and the Republic of Korea have implemented vaccination campaign [11]. Nationwide vaccination efforts contributed to the rapid containment of the first outbreak in the Republic of Korea in 2023 [16, 54]. In Iran, an epidemiological study of the 2014–2016 LSD outbreak showed that vaccination significantly reduced the occurrence of clinical disease [55]. In Thailand, a recent analysis reported that mass vaccination led to a 78%–119% reduction in the LSD incidence [56]. In contrast, vaccination during the LSD outbreak in Japan was limited because it was not mandatory. The number of new cases declined after the start of vaccination in Fukuoka Prefecture; however, this coincided with a seasonal decline in the activity of vector insects such as stable flies due to lower temperatures. This made it difficult to assess the effectiveness of vaccination in controlling the outbreak in Japan. Given that vaccination was delayed during the early phase of the outbreak, the MAFF announced its intention to revise the Act on the Prevention of Infectious Disease in Livestock in March 2025 to make LSD vaccination mandatory in preparation for future outbreaks [57].

The virus isolated from the first case farm in Japan has a 100% genetic identity with the vaccine-like recombinant strains that have been circulating in neighboring countries since 2019 (submitted by Watanabe et al. [22]). This highlights the ongoing risk of LSDV incursion into Japan from surrounding regions. The epidemiological analysis of the first LSD outbreak in Japan revealed several characteristics of LSD that may cause large-scale outbreaks: a high transmission rate among cattle, which was estimated in this study, and the potential for long-distance transmission via cattle movements as observed during the outbreak. Raising awareness among farmers and veterinarians is critical for early detection and reporting of susceptible cases to manage the risk of future outbreaks. Preparedness control measures, such as establishing a mandatory vaccination program, implementing vector control strategies, traceability of cattle movement, and enforcement of quarantine and inspection protocols for pre and postmovement cattle, are essential. These control measures are vital to reduce the risk of reemergence and protect cattle industries from significant economic losses.

## 5. Conclusion

This study conducted a descriptive epidemiological analysis to characterize the spatial and temporal features of the first LSD outbreak in Japan in 2024 and the control measures implemented. The transmissibility of LSD in cattle was estimated using a mathematical model based on within-farm LSD transmission data. The estimation of transmission rate based on field data provides valuable insights into the transmission dynamics of LSD under natural conditions. These findings contribute to the planning and enhancement of future control measures against LSD in Japan, especially in light of its ongoing spread in Asia and the risk of future incursion.

## Figures and Tables

**Table 1:** Farms reporting lumpy skin disease during the outbreak in Japan in 2024.

Case	Prefecture	Municipality	Confirmation date	Farm type	Farm size	Number of animals with clinical onset	Cumulative incidence (%)
1	Fukuoka	Itoshima city	2024/11/06	Dairy	75	48	64.0
2	Fukuoka	Itoshima city	2024/11/06	Dairy	69	33	47.8
3	Kumamoto	Kikuchi district	2024/11/09	Dairy	63	25	39.7
4	Fukuoka	Itoshima city	2024/11/10	Mixed dairy and beef	265	12	4.5
5	Fukuoka	Itoshima city	2024/11/13	Dairy	73	11	15.1
6	Fukuoka	Itoshima city	2024/11/13	Dairy	19	9	47.4
7	Fukuoka	Itoshima city	2024/11/13	Dairy	162	31	19.1
8	Fukuoka	Itoshima city	2024/11/13	Dairy	79	4	5.1
9	Fukuoka	Itoshima city	2024/11/14	Dairy	68	13	19.1
10	Fukuoka	Itoshima city	2024/11/25	Dairy	85	4	4.7
11	Fukuoka	Itoshima city	2024/11/27	Beef	10	1	10.0
12	Fukuoka	Itoshima city	2024/11/28	Dairy	37	5	13.5
13	Fukuoka	Nakagawa city	2024/11/29	Dairy	217	5	2.3
14	Fukuoka	Itoshima city	2024/12/02	Dairy	139	2	1.4
15	Fukuoka	Itoshima city	2024/12/03	Dairy	101	1	1.0
16	Fukuoka	Fukuoka city	2024/12/03	Dairy	87	1	1.1
17	Fukuoka	Itoshima city	2024/12/05	Dairy	64	1	1.6
18	Fukuoka	Asakura city	2024/12/05	Dairy	64	12	18.8
19	Fukuoka	Itoshima city	2024/12/07	Dairy	180	1	0.6
20	Kumamoto	Kikuchi district	2024/12/08	Mixed dairy and beef	165	8	4.8
21	Fukuoka	Itoshima city	2024/12/18	Beef	28	1	3.6
22	Kumamoto	Kikuchi district	2024/12/26	Beef	120	2	1.7

## Data Availability

The data that support the findings of this study are available from the corresponding author upon reasonable request, subject to approval by the Ministry of Agriculture, Forestry and Fisheries (MAFF) and the relevant prefectural governments.

## Supporting Information

Supporting Information Figure S1: Number of farms and cattle by dairy and beef farming in Kyushu Island, Japan. Figure S2: Temperature from November 1 to December 31, 2024, in Itoshima City, Fukuoka Prefecture, and the number of farms with cases of lumpy skin disease. Red dashed horizontal lines indicate 10 and 12°C. Table S1: Number of cattle farms and animals in Kyushu Island, Japan.
