# Supplementary material for: Descriptive Epidemiological Analysis for the First Outbreak of Lumpy Skin Disease in Japan in 2024
Source: Transbound Emerg Dis. 2025 Sep 29;2025:8488125. doi: 10.1155/tbed/8488125 (PMC12500357; doi:10.1155/tbed/8488125)
Supplement: Supporting Information — Figure S1: Number of farms and cattle by dairy and beef farming in Kyushu Island, Japan. Figure S2: Temperature from November 1 to December 31, 2024, in Itoshima City, Fukuoka Prefecture, and the number of farms with cases of lumpy skin disease. Red dashed horizontal lines indicate 10 and 12°C. Table S1: Number of cattle farms and animals in Kyushu Island, Japan. [file 8488125.f1.docx]

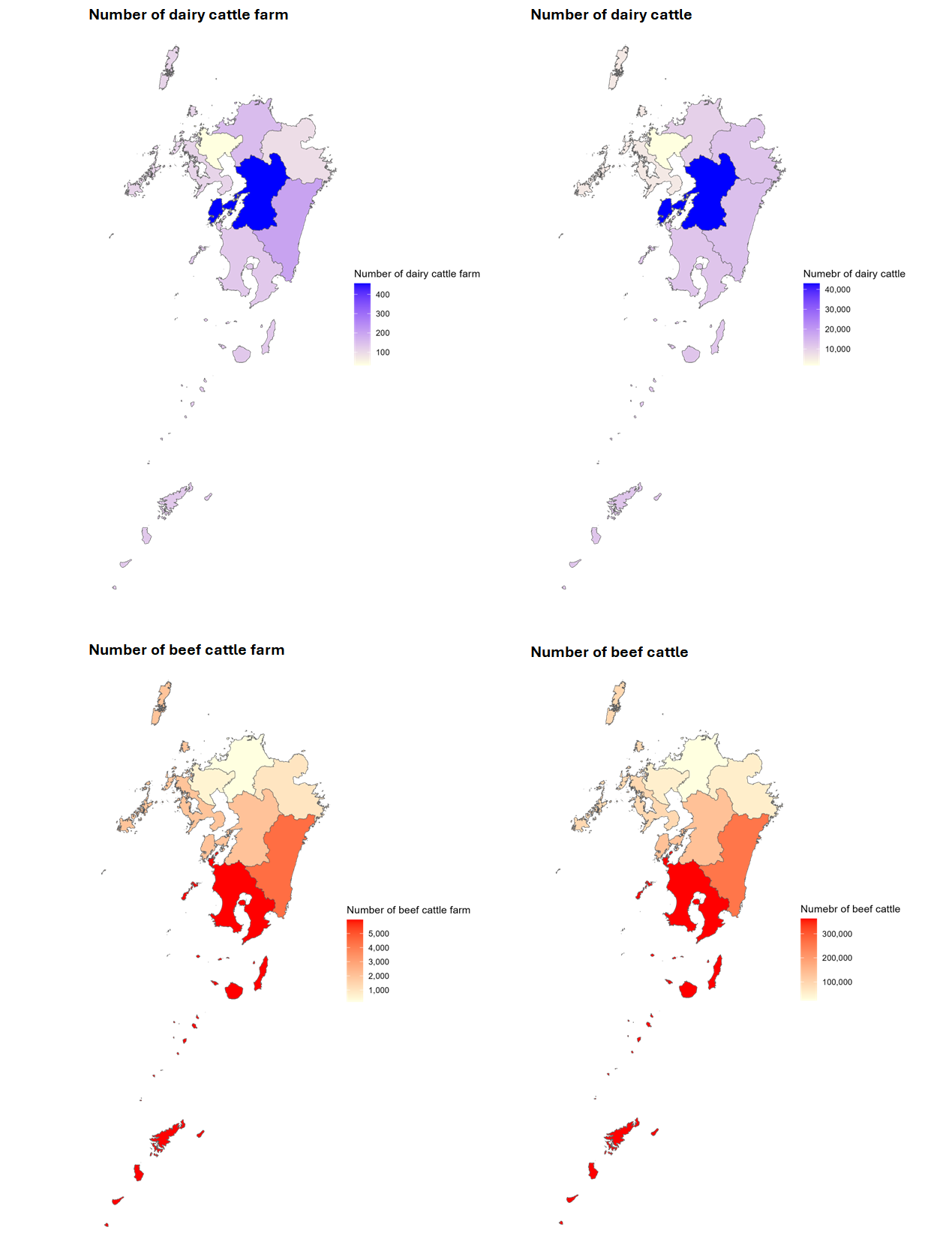


Figure S1 Number of farms and cattle by dairy and beef farming in Kyushu Island, Japan

Table S1 Number of cattle farms and animals in Kyushu Island, Japan

| Prefecture | Beef cattle farm | Beef cattle | Dairy cattle farm | Dairy cattle | Total of farm | Total of cattle |
| --- | --- | --- | --- | --- | --- | --- |
| Fukuoka | 162 | 23,200 | 154 | 10,100 | 316 | 33,300 |
| Saga | 508 | 52,200 | 32 | 1,830 | 540 | 54,030 |
| Nagasaki | 1,980 | 93,500 | 110 | 5,620 | 2,090 | 99,120 |
| Kumamoto | 2,020 | 134,000 | 457 | 43,000 | 2,477 | 177,000 |
| Oita | 964 | 54,400 | 94 | 12,300 | 1,058 | 66,700 |
| Miyazaki | 4,390 | 258,200 | 200 | 13,000 | 4,590 | 271,200 |
| Kagoshima | 5,980 | 362,700 | 131 | 12,000 | 6,111 | 374,700 |


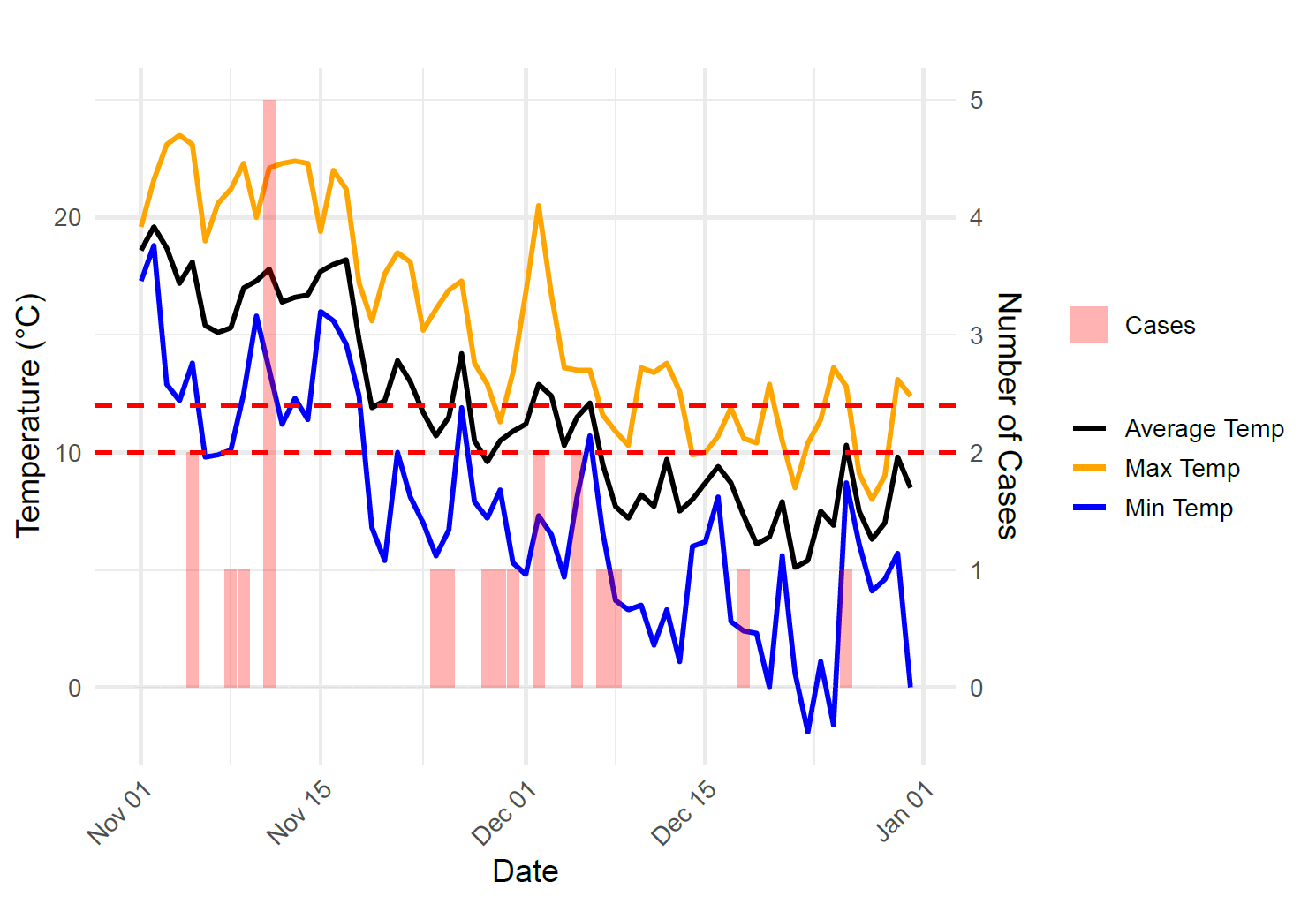


Figure 2 S Temperature from November 1 to December 31, 2024, in Itoshima City, Fukuoka Prefecture, and the number of farms with cases of lumpy skin disease. Red dashed horizontal lines indicate 10℃ and 12℃.
